# Relative Permeability Characteristics During Carbon Capture and Sequestration Process in Low-Permeable Reservoirs

**DOI:** 10.3390/ma13040990

**Published:** 2020-02-22

**Authors:** Mingxing Bai, Lu Liu, Chengli Li, Kaoping Song

**Affiliations:** 1 Department of Petroleum Engineering, Northeast Petroleum University, Daqing 163318, China; baimingxing@hotmail.com (M.B.); 18615934331@163.com (L.L.); lcl1139406976@163.com (C.L.); 2 Department of Petroleum Engineering, Xi’an Shiyou University, Xi’an 710065, China; 3 China University of Petroleum (Beijing), Beijing 102249, China

**Keywords:** carbon capture and storage, low permeability, relative permeability, miscible CO_2_ flooding

## Abstract

The injection of carbon dioxide (CO_2_) in low-permeable reservoirs can not only mitigate the greenhouse effect on the environment, but also enhance oil and gas recovery (EOR). For numerical simulation work of this process, relative permeability can help predict the capacity for the flow of CO_2_ throughout the life of the reservoir, and reflect the changes induced by the injected CO_2_. In this paper, the experimental methods and empirical correlations to determine relative permeability are reviewed and discussed. Specifically, for a low-permeable reservoir in China, a core displacement experiment is performed for both natural and artificial low-permeable cores to study the relative permeability characteristics. The results show that for immiscible CO_2_ flooding, when considering the threshold pressure and gas slippage, the relative permeability decreases to some extent, and the relative permeability of oil/water does not reduce as much as that of CO_2_. In miscible flooding, the curves have different shapes for cores with a different permeability. By comparing the relative permeability curves under immiscible and miscible CO_2_ flooding, it is found that the two-phase span of miscible flooding is wider, and the relative permeability at the gas endpoint becomes larger.

## 1. Introduction

**Figure 1:**
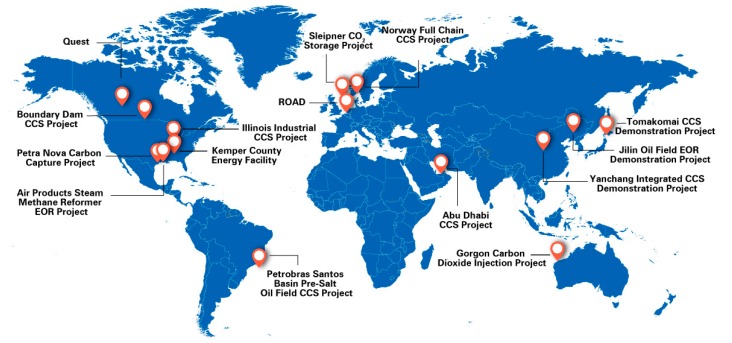
Key Carbon Capture and Sequestration (CCS) project developments and milestones.

The injection of carbon dioxide (CO_2_) in depleted oil and gas reservoirs, saline aquifers, or coal seams, is an important means of reducing air pollution and mitigating the greenhouse effect on the environment, and it has been extensively discussed in previous studies [1,2,3,4,5,6,7]. Figure 1 shows some typical Carbon Capture and Sequestration (CCS) projects around the globe. For developing countries such as China, it can serve as an important measure to enhance oil and gas recovery, especially in low- or ultralow-permeable reservoirs, since the oil in low-permeable reservoirs accounts for approximately 60% of the proven reserve which has not been developed [8]. For example, the injected CO_2_ in the ultralow-permeable reservoir in the Jilin oilfield, whose permeability is less than 0.01 × 10^−3^ μm^2^, can not only fracture the near the wellbore region and also improve the oil recovery in the reservoir.

Water flooding in low-permeable reservoirs results in a very unsatisfactory recovery, due to serious heterogeneity, tight lithology, high filtration resistance, poor pressure transmissibility and water sensitive minerals, and so on. With this regard, CO_2_ flooding is superior to water flooding, because it can not only maintain the reservoir pressure, but also improve the displacement efficiency [9,10,11]. There are several known reasons for that. For example, CO_2_ can easily dissolve into crude oil, so as to reduce the interfacial tension, the viscosity of crude oil and the residual oil saturation. In order to increase oil production and CO_2_ injection efficiency, a number of methods are used, and among them, horizontal wells play a vital role due to their large flow areas [12]. The CO_2_ flooding process in the subsurface takes place either in aquifers or sandstone reservoirs with a certain amount of oil. An important parameter for estimating the amount of oil and gas in a reservoir, and for predicting the capacity for fluid flow throughout the life of the reservoir, is the relative permeability, an indication of fluid distribution and movement, pore structure variation, and the chemistry of the solids and fluids. Moreover, all of the changes induced by the injected CO_2_ can also be observed in the relative permeability curves [13,14]. For the determination of relative permeability in low-permeable reservoirs, several concepts have to be explained. A Minimum Miscibility Pressure or MMP, the boundary of miscible flooding and immiscible flooding, shall be determined when measuring the CO_2_/oil relative permeability curve. There are several commonly-used experimental methods for the MMP determination, such as the slim-tube test, core flooding, rising-bubble apparatus, and so on [15]. In this experiment, the slim tube method is adopted. A threshold pressure, the pressure that the injected gas has to overcome to displace the fluid, has to be measured prior to the relative permeability measurement, because the complex pore structures, narrow pore throat and large tortuosity in low permeable reservoirs lead to complex physical and chemical reactions between fluid and pore walls, which cannot be described by Darcy’s law. Last but not least, the fluid viscosity has to be paid special attention during the measurement and calculation, because CO_2_, no matter whether it is miscible or immiscible, has great impact on the oil viscosity.

In this paper, the typical experimental methods and empirical correlations are reviewed and discussed with regards to the determination of relative permeability. With special focus on CO_2_ flooding, the CO_2_/water and CO_2_/oil relative permeability measurement is performed using core displacement experiment for immiscible CO_2_ flooding scenarios. Since there is little study on relative permeability in miscible flooding, the experimental method for miscible CO_2_ flooding will also be introduced.

## 2. Experimental Methods for Relative Permeability Determination

A variety of methods to measure relative permeability in porous media are basically categorized as steady-state and unsteady-state methods

### 2.1. Steady-state Methods

In steady-state methods, both phases are injected simultaneously at constant rates. Injection continues until a steady-state is reached, as indicated by a constant pressure drop and constant saturations. The subcategories of the steady-state methods include the multiple-core method, high-rate method, stationary–liquid method and uniform–capillary–pressure method, and so on. It was used to measure the relative permeability relations of supercritical CO_2_ and brine, both in imbibition and drainage processes [16,17]. However, capillary entry and end effects during two-phase flow are not considered in their work. In Chen and Amir (2014), the capillary end effect was detected during the measurement of CO_2_-brine relative permeability in a sandstone core [18]. Robin G. and Daniel R.M. (2016) introduced a novel modified steady-state approach in which corrections for end-effect artifacts are applied as data are measured [19]. However, the method process is too complex. In many studies the influence of surface tension and viscosity on the behavior of fluids is considered, but the influence of the physical state is always ignored. Florian et al. (2015) conducted the measurement of relative permeability under High Temperature and Pressure conditions with gaseous CO_2_/water and supercritical CO_2_/water with a steady-state method, and the results show that the physical state does not influence very much [20].

### 2.2. Unsteady-state Methods

In unsteady-state methods, which is also known as external-drive methods or displacement methods, only one phase is injected at either a constant flow rate or a constant pressure drop. Its subcategories include the high-rate method, low-rate method, and centrifuge methods. Unsteady-state methods are much faster than steady-state methods, but many operational difficulties are involved in unsteady-state methods, such as capillary end effects. Therefore, some modifications have been developed to improve unsteady-state methods. The Johnson–Bossier–Naumann (JBN) method, or some modification of it, interprets that high-flow-rate displacements can eliminate the capillary pressure end effects [21,22]. It consists of injecting a displacing phase into a core fully saturated with a displaced phase; during the unsteady-state portion of the displacement, both the overall pressure drop and the effluent phase ratio are measured versus time. By using fractional flow theory and a mathematical inversion, relative permeability data to both phases are obtained at the outlet as the displaced phase saturate decreases. A unique and practical method, including the capillary end effects, has been presented using sufficiently high flow rates to minimize the rate of the capillary end effects in Janos et al. (2002) [23]. However, the high-flow-rate is usually difficult to accomplish, especially in tight cores, so the end effects are often neglected in some development of simplified interpretation methods [24]. For example, a new unsteady-state model for calculating the oil–water relative permeability of low-permeability has been established, which considers the effects of threshold pressure gradient and capillary pressure [25]. In addition, a method using genetic algorithms to estimate relative permeability and minimize adjustable parameters is suggested when the capillary end-effect is high [26].

## 3. Empirical Correlations for Relative Permeability Determination

Relationships for the relative permeability as a function of saturation are usually based on experimental data. However, in the absence of data, empirical correlations are often used. The empirical correlations use the effective wetting phase saturation as the correlating parameter. In 1949, Purcell proposed an equation to compute permeability by using capillary pressure measurement. Burdine (1953) introduced a tortuosity factor into the Purcell equation, and obtained the Burdine equation (Equations (1) and (2)) [27]. 

Mualem (1976) modified the Burdine model, and the modification is known as the Mualem model (Equations (3) and (4)) [28]. This section is an excerpt from Bai et al. (2014) [29].

Burdine Model (Burdine, 1953)

(1) $$k_{rw}={\left(\frac{S_{w}-S_{wr}}{1-S_{wr}}\right)}^{2}(\int_{0}^{S_{w}}\frac{1}{P_{c}{}^{2}}dS_{w})/(\int_{0}^{1}\frac{1}{P_{c}{}^{2}}dS_{w})$$

(2) $$k_{rnw}={\left(\frac{S_{nw}-S_{wr}}{1-S_{nwr}-S_{wr}}\right)}^{2}(\int_{S_{w}}^{1}\frac{1}{P_{c}{}^{2}}dS_{w})/(\int_{0}^{1}\frac{1}{P_{c}{}^{2}}dS_{w})$$

Mualem Model (Mualem, 1976)

(3) $$k_{rw}={\left(\frac{S_{w}-S_{wr}}{1-S_{wr}}\right)}^{0.5}{\left(\int_{0}^{S_{w}}\frac{1}{P_{c}}dS_{w}\right)}^{2}/{\left(\int_{0}^{1}\frac{1}{P_{c}}dS_{w}\right)}^{2}$$

(4) $$k_{rnw}={\left(\frac{S_{nw}-S_{wr}}{1-S_{nwr}-S_{wr}}\right)}^{0.5}{\left(\int_{S_{w}}^{1}\frac{1}{P_{c}}dS_{w}\right)}^{2}/{\left(\int_{0}^{1}\frac{1}{P_{c}}dS_{w}\right)}^{2}$$

Van Genuchten (1980) has proposed the classical capillary pressures, that is, saturation relations to describe the behavior of soils. By introducing them into the Burdine model and the Mualem model, there was obtained the famous van Genuchten/Burdine model and van Genuchten/Mualem model [30,31]. In 1966 Brooks and Corey proposed the second classical capillary pressure, namely, saturation relations to describe the behavior of petroleum reservoir rock [32]. By substituting it into the Burdine model and the Mualem model with *S_nwr_* = 0, one obtains the Brooks–Corey/Burdine and the Brooks–Corey/Burdine model. In earlier experiments, Corey (1954) had obtained a linear relationship between the reciprocal of the capillary pressure squared and the effective water saturation, which is known as Corey’s correlation [33]. Corey’s correlation has been widely used, and works well, not only in the lab, but also in field applications. It is a special Brooks and Corey equation. The limitations are, for instance, that the porous media must be well-sorted, and that it is only derived for drainage process.

## 4. Relative Permeability for CO_2_ Flooding in a Low-permeable Reservoir

For a CCS project in the Jilin oilfield, a typical low–permeable reservoir, both retrieved natural cores from the reservoir and artificial cores are used to determine relative permeability in the experiment, where the unsteady-state method was adopted during immiscible CO_2_ flooding, while the steady-state method was applied during miscible CO_2_ flooding, where the evenly mixed oil and CO_2_ was injected into the cores for the relative permeability measurement. Since the miscible CO_2_ flooding is actually achieved after multi-contact and gradual extraction, even if the pressure is higher than MMP, direct gas displacement cannot ensure a completely miscible state in the cores. Thus, live oil with dissolved gas in it is used for the experiment.

### 4.1. CO_2_/Oil Relative Permeability Curve in Immiscible CO_2_ Flooding 

The unsteady-state method was adopted to conduct an immiscible CO_2_ flooding experiment based on the Buckley–Leverett theory. The immiscible flooding implies that the CO_2_ injection pressure is less than MMP of CO_2_ and oil, and CO_2_ and oil in the cores fail to reach the miscible state and are still two-phase fluid. The oil and gas flow rate versus time at the outlet of rock samples were recorded during the displacement process. Under the premise of considering the threshold pressure and gas slippage in low-permeable cores, the “JBN” method was used to calculate the oil and gas relative permeability. 

The main experimental steps include the determination of the geometric data of the core samples and the air permeability, vacuumization of the core samples and saturation with formation water, the calculation of the porosity of the rock samples, determination of the oil saturation and irreducible water saturation by displacing water with oil until no water comes out, and the measurement of oil permeability corresponding to irreducible water saturation. After this, a proper displacement pressure is applied to displace the oil by CO_2_ to ensure that the flooding velocity is between 7–30 mL/min, and the pressure is smaller than MMP. The oil and gas rate versus time is recorded during the displacement process until the residual oil saturation is reached, and then the effective permeability to gas is measured. The oil and gas flow rate measured at the outlet under atmospheric pressure is modified to the one under the average pressure in the samples, according to Equation (5). The relative permeability curves can be obtained by performing calculations according to Equation (6).

(5) $$V_{i}=\left\{ \begin{array}{l} \Delta V_{i}{}^{l}+V_{i-1}\;\;\;\;\;\;\;\;\;\;\;\;\;\;,\;\;\;\;\;\;\Delta V_{i}{}^{g}=0\\ \frac{\Delta V_{i}{}^{g}}{\alpha_{l}}\cdot E^{l}+\left(\Delta V_{i}{}^{l}-\frac{\Delta V_{i}{}^{g}}{\alpha_{l}}\right)+V_{i-1}\;\;\;\;\;\;\;,\;0<\Delta V_{i}{}^{g}<\alpha_{l}\cdot \Delta V_{i}{}^{l}\\ \Delta V_{i}{}^{l}\cdot E^{l}+V_{i-1}{}^{l}+\left[\frac{2p_{a}}{\left(\Delta p+2p_{a}\right)}\right]\cdot (\Delta V_{i}{}^{g}-\alpha_{l}\cdot \Delta V_{i}{}^{l})\;,\;\;\;\;\Delta V_{i}{}^{g}\geq \alpha_{l}\cdot \Delta V_{i}{}^{l} \end{array}\right.$$

where, *l* denotes liquid (oil or water); *V_i-1_* and *V^l^_i_* denote cumulative oil and gas rate at time *i*-1 and *i*, respectively, mL; Δ*V^l^_i_* denotes incremental liquid rate, mL*; p_a_* denotes atmospheric pressure, MPa; Δ*p* denotes differential pressure, MPa; $\alpha_{l}$ denotes solubility of CO_2_ in liquid, mL/mL; *E^l^* denotes the volume expansion coefficient of liquid, dimensionless.

(6) $$f_{l}=\frac{d{\bar{V}}_{i}{}^{l}}{d{\bar{V}}_{i}{}^{}}$$

(7) $$k_{rl}=f_{l}\frac{d[1/{\bar{V}}_{i}{}^{}]}{d[1/{\bar{V}}_{i}{}^{}\cdot I]}$$

(8) $$I=\frac{Q_{i}}{Q^{l}}\cdot \frac{\Delta P_{0}-G}{\Delta P_{i}}$$

(9) $$k_{rg}=k_{rl}\frac{\mu_{g}}{\mu_{l}}\cdot \frac{f_{g}}{f_{l}}\cdot \frac{1}{1+b/P_{ave}}$$

(10) $$b=0.0955{\left(\frac{k}{\varphi }\right)}^{-0.53}$$

(11) $$S_{g}={\bar{V}}_{i}{}^{l}-{\bar{V}}_{i}{}^{}\cdot f_{l}$$

where, *G* denotes threshold pressure, MPa; *ΔP_0_* denotes the initial pressure differential, MPa; *P_ave_* denotes the average pressure differential, MPa; *b* denotes gas slippage factor, dimensionless; *I* denotes relative injectivity, dimensionless; *f* denotes oil or water fractional content, dimensionless; *Q_0_* denotes initial liquid rate at the outlet, cm^3^/s; *Qi* denotes liquid rate at the outlet at time *I*, cm^3^/s.

For the CO_2_/oil relative permeability measurement under an immiscible state, the oil is a mixture of dead oil and kerosene, and the formation water has a Total Dissolved Solid (TDS) of 5500 mg/L. Experimental temperature is 74.9 °C and 45 °C for natural and artificial cores, respectively. The basic geometric and physical properties of the cores are listed in Table 1. The experimental scenarios are listed in Table 2. In the tables hereunder, K permeability, L length of the core, D cross-sectional area, φ porosity, P_in_ injection pressure and T temperature, respectively.

### 4.2. CO_2_/Water Relative Permeability Curve in Immiscible CO_2_ Flooding

Similar to the CO_2_/oil relative permeability, the CO_2_/water relative permeability characteristics can also be obtained by injecting CO_2_ into the water-saturating cores and recording the oil and gas rate, etc., versus time at the outlet. The “JBN” method was used to calculate the oil and gas relative permeability and corresponding saturation. For the experiment, the first step is the measurement of air permeability, saturating the rocks with water and determination of the porosity. The next step is to adjust the injection pressure by regulating the pressure valve, and to inject CO_2_ into the cores under constant pressure. The differential pressure must overcome the end effect, without leading to turbulent flow. After that, the data, such as gas breakthrough time, cumulative water and gas flow rate versus time, are all recorded. When the water saturation reaches the irreducible value, the gas permeability is measured under the displacement pressure and 1/2 displacement pressure, respectively.

The total oil and gas flow rate measured under atmospheric pressure at the outlet is modified to the one under the average pressure of the rock sample, according to Equation (5). The relative permeability curves can be obtained by performing calculations according to Equation (6).

For CO_2_/water relative permeability measurement under an immiscible state, the formation water has a TDS of 5500 mg/L. Experimental temperature is 45 °C for artificial cubic cores. The basic geometric and physical properties of the cores are listed in Table 3. The experimental scenarios are listed in Table 4.

### 4.3. CO_2_/Oil Relative Permeability Curve in Miscible CO_2_ Flooding

The steady-state method for CO_2_ miscible flooding is based on Darcy’s law without consideration of capillary pressure and gravity effect. CO_2_ and live oil are fully mixed in a certain proportion under a pressure greater than MMP to reach the miscible state. The mixture is injected into the samples under a constant pressure, while keeping the inlet and outlet pressure constant and above the MMP as well.

When the oil and gas flow at the outlet is stable, it is deemed that the distribution of oil and CO_2_ in the sample is uniform and stable. At this time the effective permeability to oil and CO_2_ is constant, and the effective permeability and relative permeability value of oil and gas can be directly calculated with Darcy’s law by using the recorded data. A series of CO_2_/oil relative permeabilities under different oil saturations can be obtained by calculating the average oil saturation with the material balance method and by changing the mixing ratio of oil and CO_2_.

The first step is to put the core samples saturated with formation water into the core holders where the water is injected through the cores with a certain flow rate via a pump. The water permeability is measured for three times after the differential pressure and flow rate at the outlet are stable. The second step is to displace the water in the samples with live oil, and to measure the effective permeability of this oil at the irreducible water saturation. The third step is to inject the CO_2_/oil mixture into the cores and record the oil and gas volume at the outlet under ambient pressure, as well as the cumulative injection volume, until the oil and gas flow is stable. In order to keep the mixture miscible, the pressure at the outlet is set to be 30 MPa. The proportion of live oil and CO_2_ in the mixture is changed, and step 3 is repeated. The effective and relative permeability of CO_2_ and oil can be calculated based on Darcy’s law.

For the CO_2_/oil miscible phase, the live oil with dissolved GOR of 30 Sm^3^/m^3^ is used. The TDS of the formation water is 5500 mg/l, and the temperature is 45 °C. The basic core data is listed in Table 5. According to the indoor experiments, the bubble point pressure is 7 MPa, the MMP of CO_2_ and live oil is 24.5 MPa, so for the four cores, the injection pressure and discharge pressure are 35 MPa and 30 MPa, respectively. The scenarios are designed in Table 6. 

## 5. Discussion of Experimental Results

### 5.1. Relative Permeability Curves for Immiscible CO_2_/Oil Displacement

The main experimental steps are described in Section 4.1. It includes mainly the determination of the oil saturation and irreducible water saturation by displacing water with oil until no water comes out, and then measurement of oil permeability corresponding to an irreducible water saturation. 

**Figure 2:**
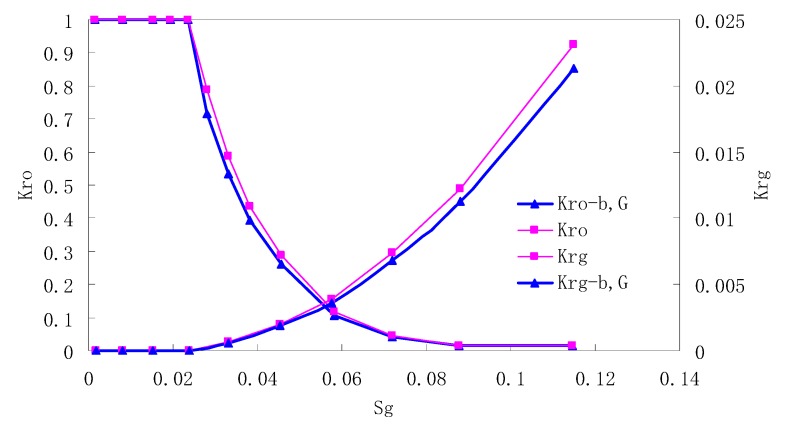
CO_2_/oil relative permeability curve for Natural Core 1 (NC1) (1.0 MPa).

**Figure 3:**
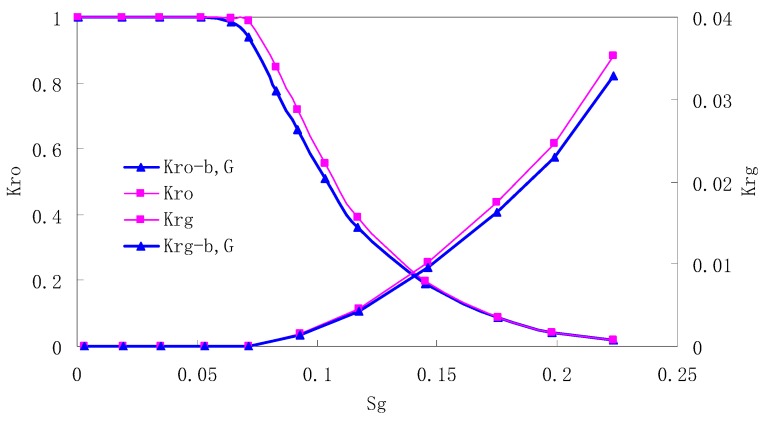
CO_2_/oil relative permeability curve for NC2 (1.0 MPa).

**Figure 4:**
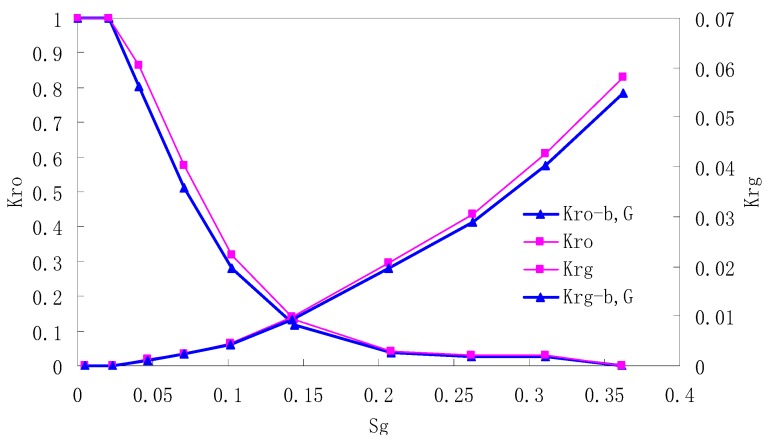
CO_2_/oil relative permeability curve for NC3 1.0 (MPa).

**Figure 5:**
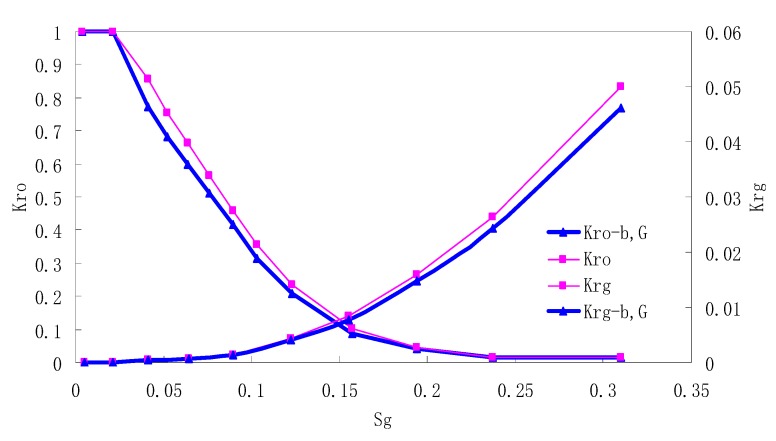
CO_2_/oil relative permeability curve for NC4 (1.0 MPa).

**Figure 6:**
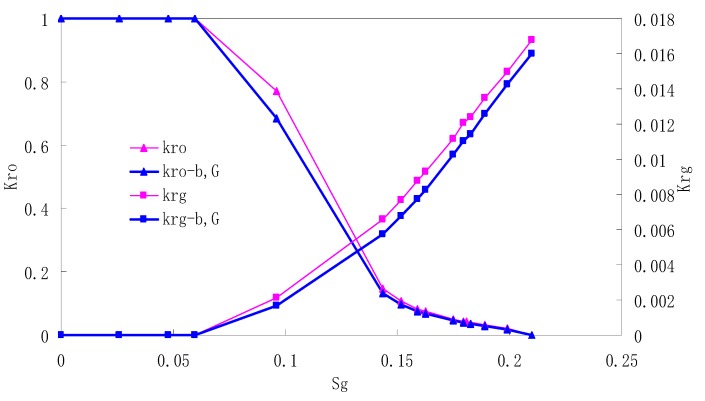
CO_2_/oil relative permeability curve for AC1.

**Figure 7:**
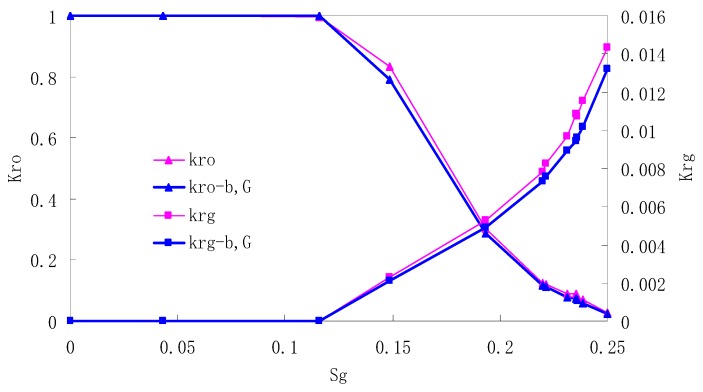
CO_2_/oil relative permeability curve for AC2.

**Figure 8:**
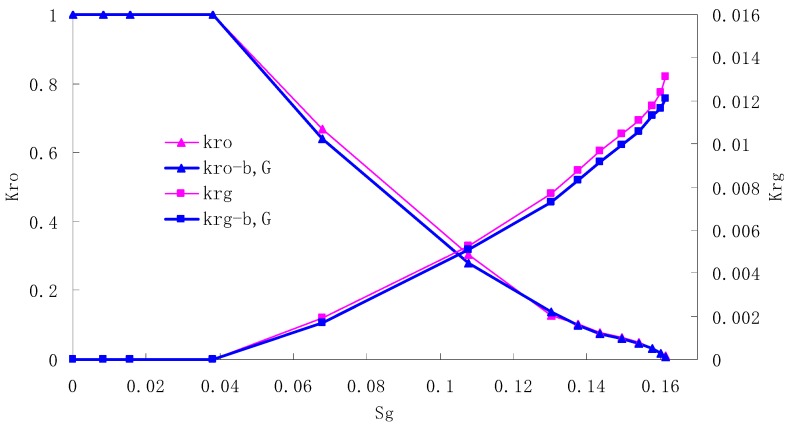
CO_2_/oil relative permeability curve for AC3.

**Figure 9:**
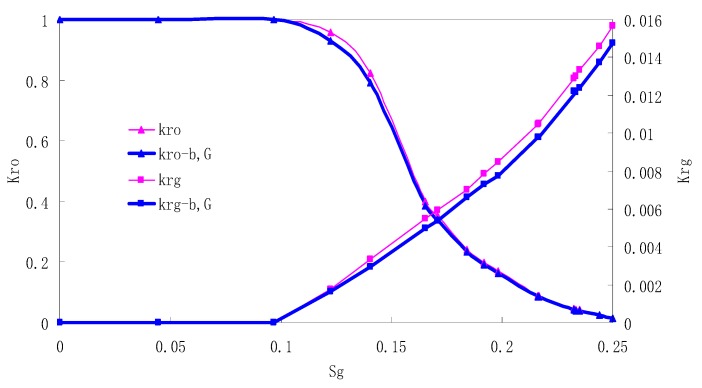
CO_2_/oil relative permeability curve for AC4.

After this, a proper displacement pressure is applied to displace the oil by CO_2_ to ensure that the flooding velocity is between 7–30 mL/min and the pressure is smaller than MMP. The relative permeability curves are obtained by performing calculations for CO_2_/oil immiscible displacement. The results are shown in Figure 2, Figure 3, Figure 4 and Figure 5 for natural cores and Figure 6, Figure 7, Figure 8 and Figure 9 for artificial cores, respectively. In the figures, K_ro-b,G_ and K_rg-b,G_ represent the relative permeability considering gas slippage and threshold pressure, while K_ro_ and K_rg_ represent relative permeability without considering them.

It can be observed from the Figures that both K_ro_ and K_rg_ are greater than K_ro-b, G_ and K_rg-b, G_, indicating that the relative permeability of both phases reduces when considering threshold pressure and gas slippage. But the relative permeability of oil does not reduce as much as that of CO_2_. When considering gas slippage, only the relative permeability of CO_2_ is modified, which has no influence on oil relative permeability. The decrease of the relative permeability of oil is because the energy exerted to displace oil is lowered when considering the threshold pressure in low permeable rocks, given that the total differential pressure is constant. When both the gas slippage and threshold pressure are considered, the relative permeability of CO_2_ decreases a little more than that to oil.

The situation of artificial cores is similar to that of natural cores. A typical feature is that the CO_2_ critical flow saturation of artificial cores is larger than that of natural cores as a whole. There are two reasons. Firstly, the natural rock sample is characterized by a wider distribution of pore size, complex pore structure and relatively strong heterogeneity, while the artificial cores have a uniform pore structure. Secondly, the porosity and size of artificial cubic cores are larger than that of natural cores, and the saturated oil quantity in it is relatively more, so the amount of the dissolved gas of CO_2_ in immiscible flooding is relatively greater.

The experimental data of natural and artificial cores are listed in Table 5 and Table 6. It can be observed from the table that no matter for natural core or artificial core, with decreasing permeability, the irreducible water saturation increases. Given that the injection pressure is the same (natural core), when the permeability decreases, the residual oil saturation will increase, and the range of the two-phase region will be narrower consequently.

The relative permeability of gas at the endpoint under immiscible flooding is relatively low due to the fact that irreducible water and residual oil in rock sample account for 61.81%–87.65% of pore space at the end of the experiment, thus the flowing space of gas is relatively small, which means the relative permeability value at the gas endpoint is relatively low.

### 5.2. CO_2_/Water Relative Permeability Curve Under Immiscible Phase

**Figure 10:**
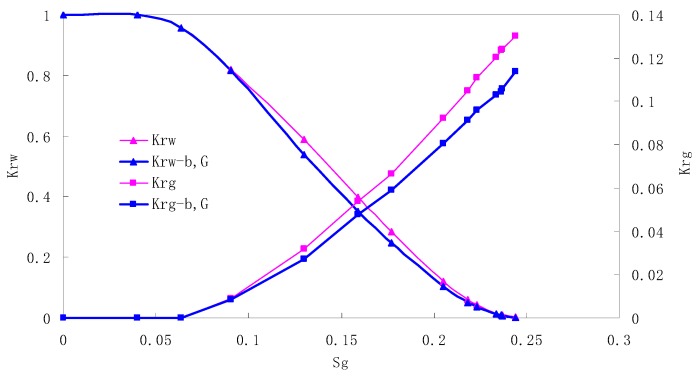
CO_2_/water relative permeability curve for AC5 (0.5 MPa).

**Figure 11:**
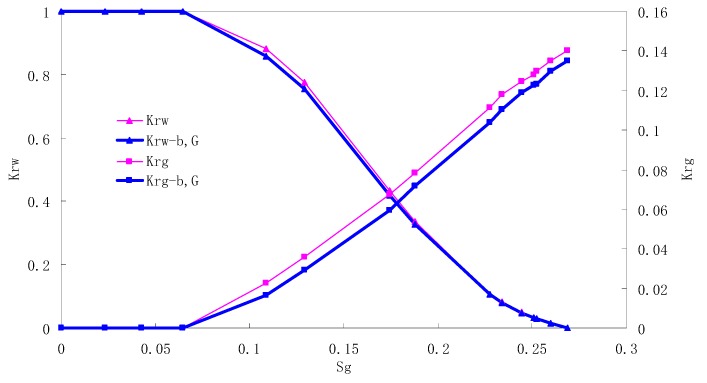
CO_2_/water relative permeability curve for AC6 (0.4 MPa).

**Figure 12:**
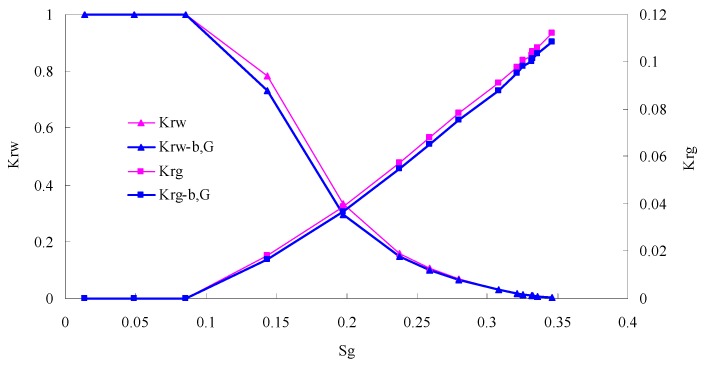
CO_2_/water relative permeability curve for AC7 (0.3 MPa).

**Figure 13:**
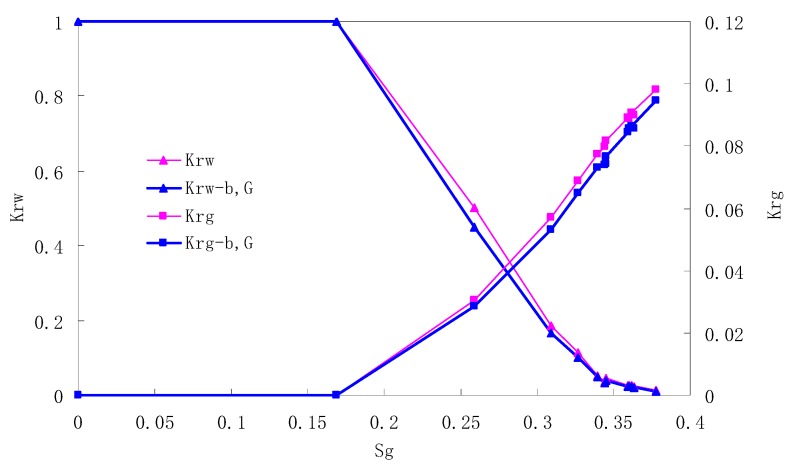
CO_2_/water relative permeability curve for AC8 (0.2 MPa).

The relative permeability curves are obtained by performing calculations for the CO_2_/water immiscible situation. The results are shown in Figure 10, Figure 11, Figure 12 and Figure 13. In the figure, K_ro-b,G_ and K_rg-b,G_ represent the relative permeability considering the solubility of CO_2_, oil viscosity change, gas slippage and threshold pressure, while K_ro_ and K_rg_ represent relative permeability without considering the gas slippage or threshold pressure.

There is critical gas saturation when considering the CO_2_ solubility. A critical saturation of gas refers to the minimum saturation at which a phase becomes mobile. In the figure, the initial section value of gas relative permeability is zero. Meanwhile, it is observed that as to the cores with similar permeability, the larger the injection pressure is, the faster the gas will breakthrough, and consequently the narrower the two-phase region will be. In the figure, both K_ro_ and K_rg_ are greater than K_ro-b, G_ and K_rg-b, G_, and it indicates that the water and gas relative permeability reduces when considering gas slippage and threshold pressure. In addition, the influence of gas slippage effect becomes smaller with the decrease of displacement pressure.

### 5.3. CO_2_/Live Oil Relative Permeability Curve under Miscible Phase

**Figure 14:**
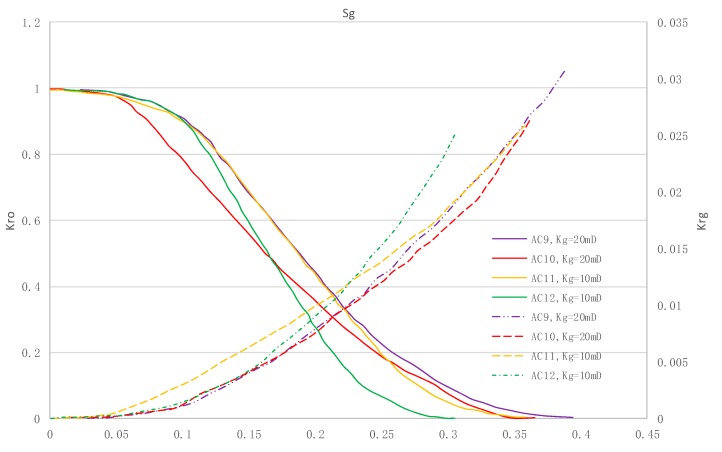
Kr curves for CO_2_ miscible flooding in AC9, AC10, AC11 and AC12 (P1 = 35 MPa).

The relative permeability curves are obtained by performing calculations for CO_2_/oil miscible situation. The results are shown in Figure 14. It can be observed from the figures that as the permeability reduces, the pore throat becomes smaller. The outcome is the decrease of the mobility of CO_2_ and oil in the cores, and the oil relative permeability decreases fast.

By comparing the kr curves under immiscible and miscible flooding situations for core samples with similar permeability, which are shown in Figure 6 through Figure 9, and Figure 14, respectively, one can find that the two-phase span of miscible flooding is wider, and the relative permeability at the gas endpoint becomes larger. In miscible flooding, the kr curves have different shapes for cores with different permeability, even under the same pressure difference. As the permeability of rock sample decreases, the gas endpoint permeability decreases, the two-phase flow region becomes narrower, and the gas saturation at the isotonic point decreases.

## 6. Conclusions and Discussions

For immiscible flooding, the relative permeability of both phases decreases when considering threshold pressure and gas slippage, and the relative permeability of oil does not reduce as much as that of CO_2_. The relative permeability of gas phase at the endpoint is relatively low. The larger the injection pressure is, the faster the gas will breakthrough, and consequently the narrower the two-phase region will be.

In miscible CO_2_ flooding, the relative permeability curves have different shapes for cores with different permeability, even under the same pressure difference. As the permeability of the rock sample decreases, the gas endpoint permeability decreases, the two-phase flow region becomes narrower, and the gas saturation at the isotonic point decreases.

By comparing the kr curves under immiscible and miscible flooding situations, one can find that the two-phase span of miscible flooding is wider, and the relative permeability at the gas endpoint becomes larger.

## Figures and Tables

**Table 1:** Geometric and physical properties of cores for immiscible CO_2_/oil displacement (NC denotes Natural Core, AC Artificial Core).

	NC1	NC2	NC3	NC4	AC1	AC2	AC3	AC4
K (× 10^−3^ μm^2^)	0.4414	0.5485	2.7186	1.0253	10.15	19.86	10.78	20.31
L (cm)	26.5	27.6	28.2	27.3	30	30	30	30
D (cm)	2.52	2.52	2.52	2.52	—	—	—	—
A (cm^2^)	—	—	—	—	4.5 × 4.5	4.5 × 4.5	4.5 × 4.5	4.5 × 4.5
φ (%)	13.02	13.07	15.52	14.38	15.31	19.25	15.83	17.74

**Table 2:** Experiment scenarios design for immiscible CO_2_/oil displacement.

	NC1	NC2	NC3	NC4	AC1	AC2	AC3	AC4
P_in_ (MPa)	1.0	1.0	1.0	1.0	1.1	0.5	1.1	0.5
T (°C)	74.9	74.9	74.9	74.9	45	45	45	45

**Table 3:** Geometric and physical properties of cores for CO_2_ displacing water.

	AC5	AC6	AC7	AC8
K (× 10^−3^ μm^2^)	20	19	19	21
L (cm)	30	30	30	30
A (cm^2^)	4.5 × 4.5	4.5 × 4.5	4.5 × 4.5	4.5 × 4.5
φ (%)	15.92	15.20	15.42	17.77

**Table 4:** Experiment scenarios design for CO_2_ displacing water.

	AC5	AC6	AC7	AC8
P_in_ (MPa)	0.5	0.4	0.3	0.2
T (°C)	45	45	45	45

**Table 5:** Geometric and physical properties of cores for the miscible CO_2_ displacement.

	AC9	AC10	AC11	AC12
K (× 10^−3^ μm^2^)	19.76	21.23	9.89	10.45
L (cm)	30	30	30	30
A (cm^2^)	4.5 × 4.5	4.5 × 4.5	4.5 × 4.5	4.5 × 4.5
φ (%)	16.84	17.23	15.62	15.73

**Table 6:** Experiment scenarios design for the miscible CO_2_ displacement.

	AC9	AC10	AC11	AC12
P_in_ (MPa)	35	35	35	35
P_out_ (MPa)	30	30	30	30
T (°C)	45	45	45	45

## References

[1] Bai M., Song K., Li Y., Sun J., Reinicke K.M. (2014). Development of a novel method to evaluate well integrity during CO_2_ underground storage. SPE J..

[2] Bai M., Sun J., Song K., Reinicke K.M., Li L., Qiao Z. (2015). Well completion and integrity evaluation for CO_2_ injection wells. Renew. Sustain. Energy Rev..

[3] Bai M., Sun J., Song K., Reinicke K.M., Teodoriu C. (2015). Evaluation of mechanical well integrity during CO_2_ underground storage. Environ. Earth Sci..

[4] Lai X.J., Ye Z.H., Xu Z.Z., Husar H.M., Henry L.W. (2012). Carbon capture and sequestration (CCS) technological innovation system in China: Structure, function evaluation and policy implication. Energy Policy..

[5] Harrison B., Falcone G. (2014). Carbon capture and sequestration versus carbon capture utilization and storage for enhanced oil recovery. Acta Geotechnica..

[6] Shaw J., Bachu S. (2002). Screening, evaluation, and ranking of oil reservoirs suitable for CO_2_-flood EOR and carbon dioxide sequestration. J. Can. Pet. Technol..

[7] Jahangiri H.R., Zhang D. (2012). Ensemble based co-optimization of carbon dioxide sequestration and enhanced oil recovery. Int. J. Greenh. Gas Control..

[8] Li C., Li H., Yin X.Q. Application of CO_2_ miscible flooding on Gao 89-1 low permeability reservoir. Proceedings of the SPE Asia Pacific Oil and Gas Conference and Exhibition.

[9] Luo P., Meng Y., Shu S., Tang L. Some problems in the exploration and exploitation low permeability of oil and gas resources in China. Proceedings of the SPE International Oil and Gas Conference and Exhibition in China.

[10] Li F., Yang S., Chen H., Zhang X., Nie X., Ding J., Zheng A. (2014). Long core physical simulation for CO_2_ flooding in low permeability reservoir. Int. J. OilGas Coal Technol..

[11] Bai M., Zhang Z., Fu X. (2016). A review on well integrity issues for CO_2_ geological storage and enhanced gas recovery. Renew. Sustain. Energy Rev..

[12] Czarnota R., Stopa J., Janiga D., Kosowski P., Wojnarowski P. (2018). Semianalytical horizontal well length optimization under pseudosteady-state conditions. Proceedings of the 2018 2nd International Conference on Smart Grid and Smart Cities (ICSGSC).

[13] Al-Wahaibi Y.M., Muggeridge A.H., Grattoni C.A. Experimental and numerical studies of gas/oil multicontact miscible displacements in homogeneous porous media. Proceedings of the 2005 SPE Reservoir Simulation Symposium held in Houston.

[14] Al-Wahaibi Y.M., Muggeridge A.H., Grattoni C.A. Gas-oil non-equilibrium in multicontact miscible displacements within homogeneous porous media. Proceedings of the 2006 SPE/DOE Symposium on Improved Oil Recovery held in Tulsa.

[15] Zhang K., Jia N., Zeng F., Li S., Liu L. (2019). A review of experimental methods for determining the Oil-Gas minimum miscibility pressures. J. Pet. Sci. Eng..

[16] Krevor S.C.M., Pini R., Zuo L., Benson S.M. (2012). Relative permeability and trapping of CO_2_ and water in sandstone rocks at reservoir conditions. Water Resour Res..

[17] Akbarabadi M., Piri M. (2013). Relative permeability hysteresis and capillary trapping characteristics of supercritical CO_2_/brine systems: An experimental study at reservoir conditions. Adv. Water Resour..

[18] Chen X.Y., Amir K. An experimental study of CO2-brine relative permeability in sandstone. Proceedings of the SPE Improved Oil Recovery Symposium held in Tulsa.

[19] Robin G., Daniel R.M. (2016). Intercept method—A novel technique to correct steady-state relative permeability data for capillary end effects. SPE Reserv. Eval. Eng..

[20] Florian O., Antonin F., Teddy F., Jean-Michel P., Arnault L., Patrick D. (2015). Experimental investigation of the influence of supercritical state on the relative permeability of Vosges sandstone. Comptes Rendus Mec..

[21] Civan F., Donaldson E.C. (1989). Relative permeability from unsteady-state displacements with capillary pressure included. SPE Form. Eval..

[22] Zhang Y., Nishizawa O., Park H., Kiyama T., Xue Z. (2017). Relative permeability of CO_2_ in a low-permeability rock: Implications for CO_2_ flow behavior in reservoirs with tight interlayers. Energy Procedia.

[23] Janos T., Tibor B., Peter S., Faruk C. (2002). Convenient formulae for determination of relative permeability from unsteady-state fluid displacements in core plugs. J. Pet. Sci. Eng..

[24] Janos T., Tibor B., Peter S., Faruk C. Direct determination of relative permeability from nonsteady-state constant pressure and rate displacements. Proceedings of the SPE Production and Operations Symposium held in Oklahoma City.

[25] Yang Y., Li X., Wu K., Lin M., Shi J. (2012). A new unsteady-state model for calculating oil-water relative permeability of low-permeability/ultra-low permeability reservoirs. Adv. Mater. Res. Vols..

[26] Ashrafi M., Helalizadeh A. (2014). Genetic algorithm for estimating relative permeability and capillary pressure from unsteady-state displacement experiments including capillary end-effect. Energy Sources Part A..

[27] Burdine N.T. (1953). Relative permeability calculations from pore size distribution data. Trans. Am. Inst. Min. Metall. Eng..

[28] Mualem Y. (1976). A new model for predicting the hydraulic conductivity of unsaturated porous media. Water Resour. Res..

[29] Bai M., Reinicke K.M., Song K. (2016). Relative permeability model and CO_2_ leakage through abandoned wells during CO_2_ underground storage. Oil Gas-Eur. Mag..

[30] Van Genuchten M.T. (1980). A closed-form equation for predicting the hydraulic conductivity of unsaturated soils. Soil Sci. Soc. Am. J..

[31] Parker J.C., Lenhard R.J., Kuppusamy T. (1987). A parametric model for constitutive properties governing multiphase flow in porous media. Water Res Res..

[32] Brooks R.H., Corey A.T. (1966). Properties of porous media affecting fluid flow. J. Irrig. Drain. Div. Proc. Am. Soc. Civil Eng..

[33] Corey A.T. (1954). The interrelation between gas and oil relative permeabilities. Prod. Mon..

